# Ecological Barriers for an Amphibian Pathogen: A Narrow Ecological Niche for *Batrachochytrium salamandrivorans* in an Asian Chytrid Hotspot

**DOI:** 10.3390/jof9090911

**Published:** 2023-09-08

**Authors:** Dan Sun, Gajaba Ellepola, Jayampathi Herath, Madhava Meegaskumbura

**Affiliations:** 1 Guangxi Key Laboratory for Forest Ecology and Conservation, College of Forestry, Guangxi University, Nanning 530000, China; sundan0991@163.com (D.S.);; 2 Department of Zoology, Faculty of Science, University of Peradeniya, Peradeniya, Kandy 20400, Sri Lanka; 3 School of Biomedical Sciences, International Institute of Health Sciences (IIHS), No. 704 Negombo Road, Welisara 71722, Sri Lanka

**Keywords:** amphibian decline, *Batrachochytrium*, chytrid pathogens, Asia, niche modelling, temperature, geographic distribution, seasonality

## Abstract

The chytrid fungal pathogens *Batrachochytrium salamandrivorans* (*Bsal*) and *B. dendrobatidis* (*Bd*) are driving amphibian extinctions and population declines worldwide. As their origins are believed to be in East/Southeast Asia, this region is crucial for understanding their ecology. However, *Bsal* screening is relatively limited in this region, particularly in hotspots where *Bd* lineage diversity is high. To address this gap, we conducted an extensive *Bsal* screening involving 1101 individuals from 36 amphibian species, spanning 17 natural locations and four captive facilities in the biodiversity-rich Guangxi Zhuang Autonomous Region (GAR). Our PCR assays yielded unexpected results, revealing the complete absence of *Bsal* in all tested samples including 51 individuals with *Bd* presence. To understand the potential distribution of *Bsal*, we created niche models, utilizing existing occurrence records from both Asia and Europe. These models estimated potential suitable habitats for *Bsal* largely in the northern and southwestern parts of the GAR. Although *Bsal* was absent in our samples, the niche models identified 10 study sites as being potentially suitable for this pathogen. Interestingly, out of these 10 sites, *Bd* was detected at 8. This suggests that *Bsal* and *Bd* could possibly co-exist in these habitats, if *Bsal* were present. Several factors seem to influence the distribution of *Bsal* in Asia, including variations in temperature, local caudate species diversity, elevation, and human population density. However, it is climate-related factors that hold the greatest significance, accounting for a notable 60% contribution. The models propose that the specific climatic conditions of arid regions, primarily seen in the GAR, play a major role in the distribution of *Bsal*. Considering the increased pathogenicity of *Bsal* at stable and cooler temperatures (10–15 °C), species-dependent variations, and the potential for seasonal *Bd*-*Bsal* interactions, we emphasize the importance of periodic monitoring for *Bsal* within its projected range in the GAR. Our study provides deeper insights into *Bsal*’s ecological niche and the knowledge generated will facilitate conservation efforts in amphibian populations devastated by chytrid pathogens across other regions of the world.

## 1. Introduction

Amphibians worldwide face considerable threats, including habitat loss, climate change, and disease, which contribute to severe population declines and biodiversity loss [1]. Chytridiomycosis, a disease caused by the chytrid fungi *Batrachochytrium dendrobatidis* (*Bd*) and *B. salamandrivorans* (*Bsal*), is a major driver of these declines [2,3,4]. Both *Bd* and *Bsal* are thought to have originated in Asia, with endemic *Bd* lineages present in the region [5,6]. *Bd* affects all three amphibian orders and has caused widespread population declines, particularly in anuran species across all amphibian-inhabited continents, and except in Asia [4,7,8]. In contrast, *Bsal* mainly affects caudates and several anurans [9,10]. It has led to severe declines in native salamanders only in Europe so far, although the pathogen is present in Asia [4,5,6,11]; however, the impending threat for the highly diverse North American and Neotropical salamanders is significant [12,13,14,15,16,17,18]. Knowledge of *Bsal* ecology is critical as it provides proactive information for amphibian conservation, including identifying new susceptible species and analyzing their occurrences with the identification of the relevant ecological drivers.

Although *Bsal* primarily infects caudates, recent studies indicate that anurans can also act as reservoir hosts or as vectors for *Bsal* [9,19,20,21,22]. This role increases *Bsal* dispersal pathways as well as disease risk to sensitive salamander species and populations as some of these species tend co-occur with anurans in the same habitat. Therefore, identifying reservoir hosts is crucial for understanding infection dynamics and potential occurrences as *Bsal* continues to expand its range and threaten biodiversity [23,24,25].

*Bsal* tends to co-occur with *Bd* in several microhabitats [19,26]. These *Bd*–*Bsal* co-occurrences perhaps facilitate hybridization, leading to the generation of new genotypes with heightened pathogenicity [11,27,28]. In their overlapping natural habitats, three species of caudata—*Salamandra salamandra*, *Triturus cristatus*, and *Ichthyosaura alpestris*—have been found to carry concurrent infections of *Bd* and *Bsal* [19,29]. It has also been demonstrated that these concurrent infections tend to escalate their severity under the specific conditions [30,31]. Additionally, *Bsal* has been found to be more pathogenic than *Bd* within the same salamander host species, which results in more severe infection outcomes [32].

Although *Bsal* has its origin in Asia, our understanding of its infections within this region is still relatively limited. Significantly, *Bsal* has been observed cohabiting with the global pandemic lineage of *Bd*, also known as *Bd*GPL, in Southeast Asia, specifically Vietnam [26]. Certain areas within Asia, such as South China, harbor multiple *Bd*-Asian genotypes along with *Bd*GPL [33,34,35]. Yet, we still do not know whether these genotypes coexist with *Bsal*. Therefore, conducting surveys on the co-occurrences of *Bsal* and *Bd* lineages in their natural habitats will yield important insights into the interactions of pathogenic chytrids.

In addition, in terms of the niche space and geographic distribution of *Bsal*, ecological niche models can be useful in predicting the potential suitable habitats of pathogens and evaluating the different influences of variables on pathogen occurrences [36]. Prior work has predicted the potential distribution of *Bsal* in Asia, with diurnal temperature range identified as the most important factor [15,37], but they failed to consider biotic interactions that might alter the range estimate. For example, alterations in biodiversity significantly impact pathogen dynamics through mechanisms such as dilution and amplification effects [38,39]. This suggests that biodiversity could play a crucial role in shaping the distribution patterns of *Bsal*. On the other hand, it is indicated that landscape structure and anthropogenic influence can further drive the dispersal risk of *Bsal* [40]. Given that human-related factors and biotic interactions significantly influence pathogen/parasite occurrences [41,42,43,44], adding biotic factors and landscape features can improve the accuracy of estimated *Bsal* distributions and niche spaces.

To identify the potential reservoir hosts and test whether *Bsal* co-occurs together with *Bd* populations in microhabitats in South China, we screened for *Bsal* infection in a regional amphibian hotspot, the Guangxi Zhuang Autonomous Region (GAR), which is newly recognized for its diverse *Bd* genotypes spanning various natural habitats [35], including where *Bsal* was previously verified [45]. To assess the influence of combined environmental factors and biotic interactions on *Bsal* occurrences in the region, we predicted the potential distribution of this pathogen through ecological niche models that included climate, landscape, and biotic characteristics. Finally, we examined the areas in the GAR that are suitable for *Bsal*. The knowledge generated that is relevant to *Bsal* ecology could advance our understanding of *Bsal* pathogenicity and disease dynamics which could have significant implications for amphibian conservation.

## 2. Materials and Methods

### 2.1. Survey Region

**Figure 1:**
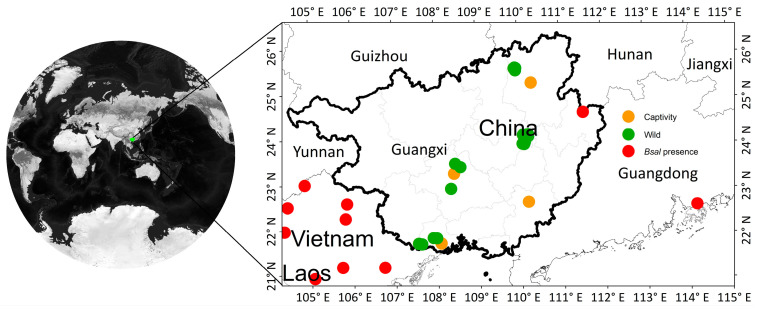
Study region and site distribution. Green points represent natural populations; orange points are for sampled captive individuals; red points indicate sites where there is a known presence of *Bsal*.

We performed *Bsal* screening throughout South China’s Guangxi Zhuang Autonomous Region (GAR) (Figure 1), which shares its southwestern border with Vietnam, a country known to harbor *Bsal* [26] from where one location was previously reported for the presence of *Bsal* [45]. The presence of *Bsal* also has been confirmed in the adjacent Guangzhou province [45]. The closest record of *Bsal* presence is located at a distance of ca. 100 km of one studied site in the GAR. In the survey region, *Bsal* has so far been detected in an individual of a salamander species, *Pachytriton wuguanfui* [45], and it is also now known that basal Asian and global lineages of *Bd* exist [35].

### 2.2. Sample Collection

To test whether *Bsal* co-occurs with *Bd* in the same habitats and *Bsal* co-infects with *Bd* in the same hosts in the surveyed region, we used previously extracted DNA from skin swabs (*N* = 1006) from amphibian adults collected between 2019 and 2021 across seventeen natural sites including nine sites confirmed for *Bd* presence [35]. These samples included 372 individuals collected in spring (April), 769 in summer (May, June, and July), 40 in autumn (October), and 12 in winter (November). Given that *Bsal* can occasionally spill over from captive populations into wildlife communities [46,47,48], we also tested the collected 95 skin swabs from a pet market and three frog farms between 2019 and 2020 and then extracted the genomic DNA from the skin swabs using PrepMan Ultra (Life Technologies, Warrington, United Kingdom) [49].

### 2.3. Detection of Bsal

We used nested PCR to detect *Bsal* on DNA extracts from skin swabs [50,51]. For the first amplification, the primers ITS1f and ITS4 were used, which specifically combined the 18S and 28S rRNA genes. The PCR amplification conditions, 4 min at 94 °C, followed by 30 cycles of 30 s at 94 °C, 30 s at 55 °C, 1 min at 72 °C, and a final 10 min at 72 °C were implemented. For the second amplification, the specific primers (STerF and STerR) for *Bsal* were used to amplify a fragment gene of the ITS-5.8S rRNA region [3]. The conditions of PCR amplification included 4 min at 94 °C, followed by 30 cycles of 30 s at 94 °C, 60 °C for 30 s, 1 min at 72 °C, and a final 10 min at 72 °C. PCR amplification products were visualized using 1.5% agarose gel electrophoresis. We used the synthetic DNA sequences of *Bsal* as positive controls and two negative controls in each plate. If PCR products yielded positive results, Sanger sequencing was conducted to verify if the amplified DNA fragments were indeed associated with *Bsal*. The methods for detection of *Bd* and its results have been described in [35].

For the individual samples detected with *Bd* infection based on nested PCR assays, we also used the simplex real-time PCR method described by [52] to test whether they were infected with *Bsal*. The real-time PCR assays were performed with the ViiA^TM^ 7 Real-Time PCR System (Life Technologies, Woodlands, Singapore) using amplification conditions consisting of 3 min at 94 °C followed by 40 cycles of denaturation at 94 °C for 5 s and annealing/extension at 60 °C for 90 s. We used duplicate samples, two negative controls, and a series of plasmid dilution standards ranging from 1.68 × 10^0^ to 1.68 × 10^6^ Molecules/μL in the real-time PCR run (Pisces Molecular, Boulder, CO, USA).

### 2.4. Ecological Niche Modelling

We used verified presence records for *Bsal* in both Europe and Asia to build the ecological niche model. Previous presence records of *Bsal* were attained from [19,26,45,53,54]. A single coordinate for each grid cell was retained to trim duplicate observation records. We used the ecoregions of *Bsal* occurrences as background to improve model calibration [55].

A total of 29 abiotic and biotic variable layers were obtained and resampled to the spatial resolution of 30 s (Table 1), and 19 bioclimatic variables represented the current climatic conditions. The human footprint that imposed pressure on the environment [56] and the human population density indicated the potential distribution of *Bsal* mediated by humans. The normalized difference vegetation index (NDVI), the enhanced vegetation index (EVI), and the net primary productivity (NPP) represented the natural state of the habitats. We used the maximum of monthly NDVIs and EVIs to synthesize the yearly NDVIs and EVIs. The average yearly NDVIs, EVIs, and NPP from 2019, 2020, and 2021 were used as the final predictor variables to reduce the potential inter-annual variation. The amphibian species’ richness and caudate species’ richness were used as biotic information to improve model fit and better understand the relationships between the species community and *Bsal* occurrences. To exclude the effects of high collinearity between predictors, we calculated the correlations between variables in the ENMTools [57], then retained eleven variables with Pearson’s r < 0.7 to construct ecological niche models (Tables S1 and S2).

We applied two model classes: generalized liner model (GLM) and Maximum Entropy Modeling (MaxEnt) to evaluate the relative importance influencing *Bsal* occurrences and to estimate the suitable habitats of *Bsal* [58,59]. The model performance was evaluated using the cross-validated area under the receiver (AUC) and true skill statistic (TSS) [60,61], which was calculated by splitting the training (70%) and testing (30%) observations. AUC values below 0.7 were poor, 0.7–0.9 were good, and 0.9–1.0 were excellent [62,63]. TSS values below 0.4 were poor, 0.4–0.8 were useful, and >0.8 were good to excellent [64]. GLM and Maxent models were performed in “sdm” packages [65], with 10 replications for each model. The ensemble model was generated using weighted averaging based on TSS statistics and the threshold of maximizing the sum of sensitivity and specificity [66]. We finally extracted the studied region from generated *Bsal* distribution maps. All analyses in this study were performed in ArcGIS 10.4.1 (Environmental Systems Research Institute, 16th June 2021) and R 4.2.2 (R Development Core Team, 31th October 2022).

**Table 1:** All initial predictor variables, their sources, and the related biological hypotheses. Bold fonts indicate final predictor variables used in our ecological niche models, with Pearson’s r < 0.7. These predictor variables represent three main categories including climate, landscape (non-climate), and biotic factors, and the corresponding hypotheses for the associations of these factors to *Bsal* presence.

	Variable	Hypotheses	Source
Climate factor	Annual mean temperature (bio_1)	Temperature and moisture affect *Bsal* life history and pathogenicity [3,22,67].	WorldClim v.2.1 [68]
	**Mean diurnal temperature range (bio_2)**		
	Isothermality (bio_3)		
	Temperature seasonality (bio_4)		
	Max temperature of warmest month (bio_5)		
	Min temperature of coldest month (bio_6)		
	**Annual temperature range (bio_7)**		
	Mean temperature of wettest quarter (bio_8)		
	**Mean temperature of driest quarter (bio_9)**		
	Mean temperature of warmest quarter (bio_10)		
	Mean temperature of coldest quarter (bio_11)		
	**Annual precipitation (bio_12)**		
	Precipitation of wettest month (bio_13)		
	Precipitation of driest month (bio_14)		
	Precipitation seasonality (bio_15)		
	Precipitation of wettest quarter (bio_16)		
	Precipitation of driest quarter (bio_17)		
	Precipitation of warmest quarter (bio_18)		
	**Precipitation of coldest quarter (bio_19)**		
Landscape factor	**Altitude**	It is expected that these factors have an important influence on *Bsal* distribution, with human-related factors having positive relationships with probabilities of *Bsal* occurrence.	http://srtm.csi.cgiar.org/srtmdata/ (accessed on 22 June 2023)
	Soil water stress		[69]
	Human footprint		[56]
	**Human population density**		[70]
	Normalized difference vegetation index		https://www.earthdata.nasa.gov/ (accessed on 26 June 2023)
	**Enhanced vegetation index**		https://www.earthdata.nasa.gov/ (accessed on 26 June 2023)
	**Net primary productivity**		https://www.earthdata.nasa.gov/ (accessed on 26 June 2023)
Biotic factor	**Amphibian species’ richness**	Dilution or amplification effects of biodiversity have profound influence on pathogen transmission [38,71]. Higher amphibian species richness might pose dilution effect since it possibly includes resistant species, whereas higher caudate species richness may pose an amplification effect since it may include more susceptible species.	[72]
	**Caudate species’ richness**		[72]

## 3. Results

### 3.1. Bsal Absence in Wild and Captive Amphibians

We did not detect *Bsal* infection based on nested PCR assays in any of the individuals (*N* = 1101, representing 36 amphibian species), of which 51 individuals from 16 species were infected with *Bd* (Table 2). The individuals infected with *Bd* consisted of 48 wild individuals of 15 species and 3 captive individuals belonging to the Chinese giant salamander (*Andrias davidianus*). Also, *Bsal* was not detected with the simplex real-time PCR assay in these *Bd*-infected individuals.

### 3.2. Bsal Models

**Figure 2:**
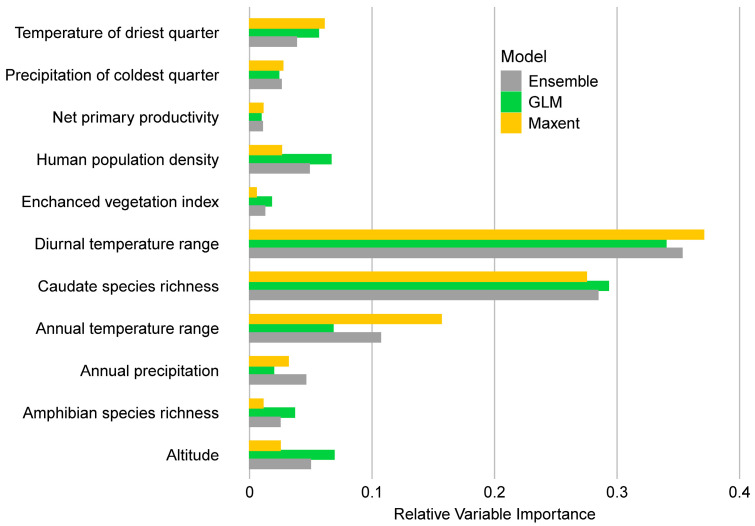
Importance of average relative variables in the final ecological niche models for *Bsal*. Importance (permutation importance) of predictor variable for GLM models, Maxent models, and ensemble models were evaluated and scaled based on the AUC metric.

The average AUC and TSS values for the *Bsal* distribution model were 0.90 (SD ± 0.04) and 0.74 (SD ± 0.08), respectively, signifying good model performance. The top two variables for model predictions included mean diurnal temperature range and caudate species richness (Figure 2). The overall contribution of the climate-related five variables formed the largest importance (60%) in explaining *Bsal* distribution. Predictor variables associated with landscape factors (i.e., net primary productivity, human population density, and enhanced vegetation index) jointly contributed towards explaining about 12% of the *Bsal* distribution, although a single landscape-related variable did not show high relative importance. Factors associated with biotic interactions significantly affected the predicted *Bsal* distribution, with caudate species occurrence being positively related to the presence of this pathogen (Figure S1).

**Figure 3:**
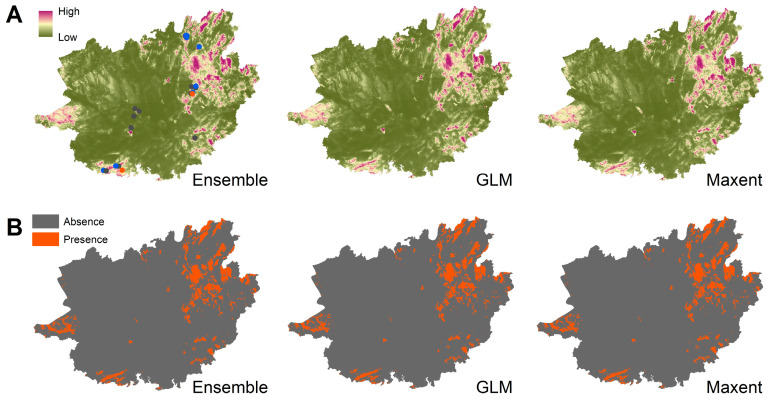
Predicted suitable habitats of *Bsal* in the studied region: (**A**) habitat suitability; (**B**) presence–absence distribution. Blue points represent the studied sites suitable for *Bsal* with confirmed *Bd* presence. Orange represents the studied sites suitable for *Bsal*, where *Bd* was not detected. Grey represents the studied sites that are unsuitable for *Bsal* occurrence.

The niche models of *Bsal* estimated that the suitable habitats for *Bsal* are in the northern and southwestern parts of the GAR (Figure 3; *Bsal* suitability range: 0.49–0.94). The challenging ecological conditions prevalent in much of the GAR, which include high fluctuations in temperature and precipitation, generally exceed the environmental preferences of *Bsal* (Table S3).

Among the 21 surveyed wild sites, 10 were deemed suitable for *Bsal*, with suitability indexes ranging from low (0.54) to high (0.89). These suitable sites were confirmed for *Bd* presence except for one frog farm and one wild site (Figure 1).

## 4. Discussion

This study provides insights into the ecological niches of the deadly amphibian pathogen *Batrachochytrium salamandrivorans* (*Bsal*) within an Asian chytrid hotspot. Despite our extensive sampling of 1101 individuals across 36 species, *Bsal* was not detected. This absence contrasts with the detection of *Bd*, which was present in 51 individuals from 16 amphibian species, including the Chinese giant salamander *A. davidianus,* known to be susceptible to both pathogens [10,45,73]. This differential distribution may indicate that these pathogens have distinct ecological niches, potentially driven by differences in their biotic or abiotic preferences.

Drawing upon presence data from extensive studies across Asia and Europe, our ecological niche models underscore the influence of certain biotic and abiotic factors in shaping the potential distribution of *Bsal*. As indicated by robust AUC and TSS values, the models provide a strong performance, pointing to the mean diurnal temperature range and caudate species richness as notable predictors. These insights suggest a unique thermal niche preference for *Bsal*, which may not be met within the habitats of the amphibians surveyed in our study. The observed absence of *Bsal* could be attributed to the scarcity of caudates in the area and the temperature-dependent pathogenicity of the organism. *Bsal* prefers lower thermal conditions for its survival; temperatures above 22 °C not only inhibit its growth but also reduce its ability to infect hosts, even those that are highly susceptible [67]. Previous research supports this observation, suggesting that temperature is a key factor behind low prevalence of *Bsal* in Asian amphibians [45]. Both the environmental and water temperatures in these regions are generally above 15 °C, which is considered suboptimal for survival and growth of *Bsal* [3,26,45,74]. Furthermore, our models reinforce earlier findings regarding the significant influence of diurnal temperature range on *Bsal* distribution [15], and highlight the contribution of biotic interactions, particularly with caudate species, in shaping the niche space of this pathogen.

The absence of confirmed cases echoes previous studies [51,75], underscoring that *Bsal* may display a transient persistence with reduced transmission rates among anurans [22]. This finding warrants a deeper investigation into host-specific factors, such as skin factors, that could be influencing the presence or detection of *Bsal* [76]. Furthermore, the influence of environmental factors such as plant materials in the water on *Bsal* growth [77] raises the possibility of *Bsal* being present in specific microhabitats [78] or reservoir hosts that were not covered in our survey. It is important to note that the sample size for tested caudates in the study is relatively small—only 30 individuals from 3 species out of a total of 1101 samples representing 36 amphibian species. This is particularly noteworthy given that salamanders are documented to be the most susceptible to *Bsal* [10,18,22]. To this end, extending the range of sampling efforts in environmental reservoirs and salamanders could enrich our understanding of the existence of *Bsal*.

Interestingly, the niche models predicted that some areas within the northern and southwestern regions of the GAR would provide suitable habitats for *Bsal*. Notably, 10 out of the 21 natural sites surveyed were deemed suitable for *Bsal*. This, coupled with the presence of *Bd* at eight of these sites, raises the possibility of a potential co-occurrence of these pathogens in these habitats, despite the current absence of *Bsal* at present. Hence, future monitoring of these habitats, especially across seasons, will be important to understand the infection dynamics of pathogens, pathogenicity, and potential resource partitioning between *Bsal* and *Bd*. Additionally, investigating how both pathogens affect the same amphibian host species (c.a. 28, [10,79], Table S4) could offer valuable insights into their potential interactions and mutual influence on infections. For instance, in an initial *Bsal* inoculation experiment involving the North American eastern newt (*Notophthalmus viridescens*), it was observed that a *Bd* infection negatively impacted the subsequent *Bsal* infection [80].

The absence of *Bsal* in habitats suitable for its presence, as suggested by the niche models, opens up further possibilities as well. While it is possible that *Bsal* has not yet been introduced to these regions, it could also be the case that the pathogen has been introduced but is not able to establish itself due to unknown ecological or biological barriers like suboptimal habitat connectivity [78,81,82]. For instance, previous studies highlight the significant role of seasonality, driven by variations in temperature and precipitation, in determining *Bsal* distribution and host susceptibility [67,83]. Considering that our sampling primarily targeted amphibians during their reproductive period in spring and summer, future surveys focusing on cooler seasons could potentially yield different results, given the known preference of *Bsal* for consecutive colder thermal conditions [3,74].

Finally, our study suggests a complex interplay of ecological and biological factors in dictating the distribution of amphibian chytrid fungal pathogens. As climate change continues to alter key ecological factors, the risk of *Bsal* is expected to persist in coastal areas and higher elevations, even though habitats with medium-to-high climatic suitability for *Bsal* are likely to decrease [37,84]. The international and regional trade in amphibians has been identified as a significant driver for the translocation of both *Bd* and *Bsal* pathogens [5,29]. Our discovery of *Bd* in *A. davidianus—*a species susceptible to *Bsal* [10]—sourced from a pet market suggests that the emergence of *Bd* in natural amphibian populations in the GAR could be linked to a spillover from captive animals, facilitated by human activities. As such, regular monitoring and surveillance programs are essential for the early detection of these pathogens, enabling timely interventions to mitigate disease risks [85]. Further research is urgently needed to understand the infection dynamics of these deadly pathogens, as this knowledge will be pivotal for predicting and managing future outbreaks among amphibian populations, both in Asia and suitable habitats across the world.

## 5. Conclusions

Our study sheds light on the ecological niches of the amphibian pathogen *Bsal* within an Asian chytrid hotspot. Despite rigorous sampling, *Bsal* was not found, but *Bd* was detected. This disparity could signal distinct ecological niches for these pathogens, possibly influenced by different biotic or abiotic factors. Our niche models suggest the importance of temperature in explaining the absence of *Bsal* in the study sites. This research reinforces the significance of diurnal temperature range on *Bsal* distribution and reveals the role of biotic interactions in shaping its niche space. The lack of *Bsal* may be due to transient persistence with reduced transmission rates; this warrants further study into host-specific factors. Interestingly, some regions of the GAR are predicted to be suitable for *Bsal*, despite its current absence, which is suggestive of potential co-occurrence with *Bd* in the future. This suggests that unknown ecological or biological barriers maybe responsible for *Bsal* establishment at present. Our research underlines the complex interplay of ecological and biological factors in determining amphibian chytrid fungal distribution and the importance of understanding these dynamics for future disease management in the GAR, and other regions of the world that *Bsal* has not yet penetrated.

## Figures and Tables

**Table 2:** Identification of species and individual for *Bsal* presence in the current study. Species highlighted in bold were previously recorded as *Bd*-positive according to [35], with the exception of *A. davidianus*, which was detected herein. Species denoted with an asterisk (*) represent samples obtained from captive amphibians.

Family	Species	Individual	*Bd*	*Bsal*
Dicroglossidae	*Fejervarya multistriata*	14	0	0
Dicroglossidae	*Hoplobatrachus chinensis*	16	0	0
Dicroglossidae	*Limnonextes bannaensis*	9	0	0
Dicroglossidae	*Quasipaa boulengeri*	34	1	0
Dicroglossidae	*Quasipaa spinosa*	66	0	0
Dicroglossidae	*Hoplobatrachus chinensis* *	15	0	0
Megophryidae	*Brachytarsophrys carinense*	2	0	0
Megophryidae	*Leptobrachella liui*	19	3	0
Megophryidae	*Leptobrachella shiwadashanensis*	7	0	0
Megophryidae	*Leptobrachium guangxiense*	2	0	0
Megophryidae	*Ophryophryne microstoma*	11	0	0
Megophrorida	*Xenophrys major*	3	0	0
Microhylidae	*Kaloula pulchra*	1	1	0
Microhylidae	*Microhyla heymonsi*	13	0	0
Microhylidae	*Microhyla pulchra*	11	0	0
Ranidae	*Amolops chunganensis*	22	13	0
Ranidae	*Amolops ricketti*	271	1	0
Ranidae	*Hylarana latouchii*	2	0	0
Ranidae	*Hylarana maosonensis*	17	0	0
Ranidae	*Hylarana guentheri*	40	0	0
Ranidae	*Odorrana exiliversabilis*	12	0	0
Ranidae	*Odorrana graminea*	53	2	0
Ranidae	*Odorrana lungshengensis*	22	6	0
Ranidae	*Odorrana nasuta*	26	0	0
Ranidae	*Odorrana versabilis*	20	3	0
Ranidae	*Rana hanluica*	4	1	0
Ranidae	*Lithobates catesbeiana* *	75	0	0
Rhacophorida	*Kurixalus odontotarsus*	42	2	0
Rhacophorida	*Liuixalus shiwandashan*	3	0	0
Rhacophorida	*Polypedates megacephalus*	131	1	0
Rhacophorida	*Rhacophorus minimus*	37	3	0
Rhacophorida	*Theloderma rhododiscus*	38	8	0
Rhacophorida	*Zhangixalus dennysi*	32	1	0
Rhacophorida	*Zhangixalus pinglongensis*	1	0	0
Salamandrida	*Pachytriton inexpectatus*	20	2	0
Salamandrida	*Pachytriton moi*	5	0	0
Salamandridae	*Andrias davidianus* (Larva) *	5	3	0

## Data Availability

All data are available within the manuscript, and supplementary information.

## Supplementary Materials

The following supporting information can be downloaded at: https://www.mdpi.com/article/10.3390/jof9090911/s1. Table S1: Pearson’s correlation of bioclimatic layers from WorldClim used in the predicted range. Table S2: Pearson’s correlation of climatic layers, landscape (non-climate), and biotic layers used in the predicted range. Table S3: Minimum, mean ± SD and maximum scores of predictor variables based on the actual *Bsal* occurrences and predicted areas of presence and absence areas for *Bsal* in the Guangxi region with ensembled models. Table S4: The 28 amphibian species that have tested positive for *Bsal* and *Bd*. Figure S1: Response of *Bsal* to predictor variables based on the ensemble models.
