# Supplementary material for: Ecological Barriers for an Amphibian Pathogen: A Narrow Ecological Niche for Batrachochytrium salamandrivorans in an Asian Chytrid Hotspot
Source: J Fungi (Basel). 2023 Sep 8;9(9):911. doi: 10.3390/jof9090911 (PMC10532633; doi:10.3390/jof9090911)
Supplement: Supplementary file 1 [file jof-09-00911-s001.zip › jof-2574541-supplementary.pdf]

Table S1. Pearson's correlation of bioclimatic layers from WorldClim used in the predicated range. See Table 1 for climatic variable abbreviations

Variable	bio_1	bio_2	bio_3	bio_4	bio_5	bio_6	bio_7	bio_8	bio_9	bio_10	bio_11	bio_12	bio_13	bio_14	bio_15	bio_16	bio_17	bio_18	bio_19
bio_1	1.00																		
bio_2	0.22	1.00																	
bio_3	0.41	0.75	1.00																
bio_4	-0.47	-0.44	-0.90	1.00															
bio_5	0.91	0.24	0.16	-0.14	1.00														
bio_6	0.93	0.15	0.54	-0.69	0.73	1.00													
bio_7	-0.31	0.05	-0.61	0.85	0.08	-0.62	1.00												
bio_8	0.81	0.10	0.14	-0.14	0.79	0.64	-0.03	1.00											
bio_9	0.71	0.33	0.59	-0.67	0.57	0.81	-0.53	0.30	1.00										
bio_10	0.92	0.05	0.07	-0.10	0.97	0.76	0.01	0.84	0.53	1.00									
bio_11	0.96	0.31	0.61	-0.70	0.78	0.98	-0.53	0.69	0.80	0.78	1.00								
bio_12	0.64	-0.10	-0.02	-0.05	0.60	0.49	-0.02	0.65	0.25	0.69	0.53	1.00							
bio_13	0.76	0.04	0.19	-0.22	0.67	0.62	-0.14	0.75	0.36	0.75	0.68	0.94	1.00						
bio_14	-0.65	-0.47	-0.63	0.60	-0.50	-0.64	0.36	-0.54	-0.53	-0.47	-0.71	-0.12	-0.40	1.00					
bio_15	0.86	0.26	0.43	-0.42	0.73	0.74	-0.24	0.82	0.49	0.77	0.82	0.70	0.87	-0.74	1.00				
bio_16	0.77	0.04	0.19	-0.23	0.67	0.63	-0.15	0.75	0.36	0.75	0.69	0.94	0.99	-0.40	0.87	1.00			
bio_17	-0.60	-0.48	-0.67	0.64	-0.42	-0.62	0.41	-0.50	-0.50	-0.39	-0.68	-0.05	-0.33	0.98	-0.69	-0.33	1.00		
bio_18	0.65	-0.09	0.09	-0.13	0.51	0.51	-0.16	0.73	0.21	0.65	0.55	0.86	0.90	-0.32	0.79	0.91	-0.27	1.00	
bio_19	-0.58	-0.48	-0.59	0.53	-0.44	-0.54	0.29	-0.60	-0.31	-0.41	-0.62	-0.11	-0.37	0.91	-0.70	-0.37	0.92	-0.35	1.00

Table S2. Pearson's correlation of climatic layers, landscape (non-climate) and biotic layers used in the predicated range. Landscape and biotic variable abbreviations: SWS: soil water stress; ASR: amphibian species richness; EVI: enhanced vegetation index; HF: human footprint; HPD: human population density; NDVI: normalized difference vegetation index; NPP: net primary productivity; CSR: Caudate species richness.

Variable	bio_2	bio_7	bio_8	bio_9	bio_12	bio_19	SWS	ASR	Altitude	EVI	HF	HPD	NDVI	NPP	CSR
bio_2	1.00														
bio_7	0.05	1.00													
bio_8	0.10	-0.03	1.00												
bio_9	0.33	-0.53	0.30	1.00											
bio_12	-0.10	-0.02	0.65	0.25	1.00										
bio_19	-0.48	0.29	-0.60	-0.31	-0.11	1.00									
SWS	0.06	0.03	0.82	0.38	0.84	-0.36	1.00								
ASR	0.17	-0.21	0.59	0.37	0.50	-0.45	0.70	1.00							
Altitude	0.45	-0.13	0.16	0.04	0.26	-0.37	0.23	0.34	1.00						
EVI	0.06	-0.16	0.15	0.01	0.23	-0.20	0.22	0.28	0.26	1.00					
HF	-0.12	0.09	-0.30	-0.20	-0.43	0.25	-0.39	-0.48	-0.61	-0.51	1.00				
HPD	-0.09	0.02	0.08	0.00	0.05	0.02	0.06	-0.05	-0.13	-0.28	0.50	1.00			
NDVI	0.06	-0.09	0.13	-0.06	0.29	-0.16	0.23	0.29	0.43	0.82	-0.71	-0.32	1.00		
NPP	0.09	-0.16	0.21	0.16	0.16	-0.19	0.19	0.06	0.10	-0.20	-0.35	0.24	-0.16	1.00	
CSR	-0.14	0.14	-0.71	-0.41	-0.59	0.54	-0.68	-0.36	-0.29	-0.15	0.36	-0.04	-0.16	-0.22	1.00

Table S3. Minimum, mean±SD and maximum scores of predictor variables based on the actual *Bsal* occurrences, predicted areas of presence and absence for *Bsal* in Guangxi region with ensembled models.

	Actual *Bsal* occurrences			Areas suitable to *Bsal*			Areas unsuitable to *Bsal*		
**Variable**	**Minimum**	**Mean± SD**	**Maximum**	**Minimum**	**Mean± SD**	**Maximum**	**Minimum**	**Mean± SD**	**Maximum**
Mean diurnal temperature range (bio_2)	4.15	7.08±0.93	10.98	5.05	7.09±0.33	8.33	5.76	7.45±0.39	8.97
Annual temperature range (bio_7)	16.2	22.02+2.98	34.2	18.8	24.55±2.46	28.5	19.2	24.18±1.98	29.6
Mean temperature of driest quarter (bio_9)	-6.02	9.03±4.69	22.28	3.18	10.48±2.84	18.17	4.75	13.31±2.54	19.28
Annual precipitation (bio_12)	664	1238.27±657.98	4294	1275	1686.97±137.77	2704	1163	1557.10±144.14	2669
Precipitation of coldest quarter (bio_19)	33	225.78±94.83	436	54	167.99±40.97	215	44	142.78±40.02	232
Amphibian species richness	0	17.30±7.61	44	0	31.86±5.83	46	0	26.38±4.61	46
Altitude	4	430.51±560.52	3133	0	788.04±366.85	1990	-1	332.07±259.05	1720
Enhanced vegetation index	0.26	0.56±0.08	0.75	0.16	0.62±0.07	0.79	0.03	0.58±0.06	0.83
Human population density	1.54	775.54±961.70	3489.05	0	447.16±2782.55	88650.05	0	196.56±263.90	8723.3
Net primary productivity	5247	11455.22±8588.38	32766	1543	10107.68±4927.30	32766	84	9480.07±4304.46	32766
Caudate species richness	0	3.82±1.80	5	0	3.09±1.48	5	0	1.44±1.38	5

Table S4. The 28 amphibian species that have tested positive for *Bsal* and *Bd*. The IUCN Red List of Threatened Species (https://www.iucnredlist.org): Least concern (LC), Near threatened (NT), Vulnerable (VU), Endangered (EN), Critically endangered (CR). NA represents no data.

**Family**	**Species**	**IUCN**
Alytidae	*Alytes obstetricans*	LC
Ambystomatidae	*Ambystoma maculatum*	LC
Ambystomatidae	*Ambystoma opacum*	LC
Cryptobranchidae	*Andrias davidianus*	CR
Lissotriton boscai	*Lissotriton boscai*	LC
Plethodontidae	*Aquiloeurycea cephalica*	NT
Plethodontidae	*Desmognathus conanti*	NA
Plethodontidae	*Eurycea cirrigera*	LC
Plethodontidae	*Eurycea guttolineata*	LC
Plethodontidae	*Eurycea lucifuga*	LC
Plethodontidae	*Pseudotriton ruber*	LC
Ranidae	*Rana temporaria*	LC
Salamandridae	*Cynops ensicauda*	EN
Salamandridae	*Cynops orientalis*	LC
Salamandridae	*Euproctus platycephalus*	EN
Salamandridae	*Ichthyosaura alpestris*	LC
Salamandridae	*Lissotriton helveticus*	LC
Salamandridae	*Lissotriton vulgaris*	LC
Salamandridae	*Notophthalmus viridescens*	LC
Salamandridae	*Pleurodeles nebulosus*	LC
Salamandridae	*Pleurodeles waltl*	NT
Salamandridae	*Salamandra salamandra*	VU
Salamandridae	*Taricha granulosa*	LC
Salamandridae	*Triturus cristatus*	LC
Salamandridae	*Triturus dobrogicus*	NT
Salamandridae	*Triturus macedonicus*	VU
Salamandridae	*Triturus marmoratus*	LC
Sirenidae	*Siren intermedia*	LC

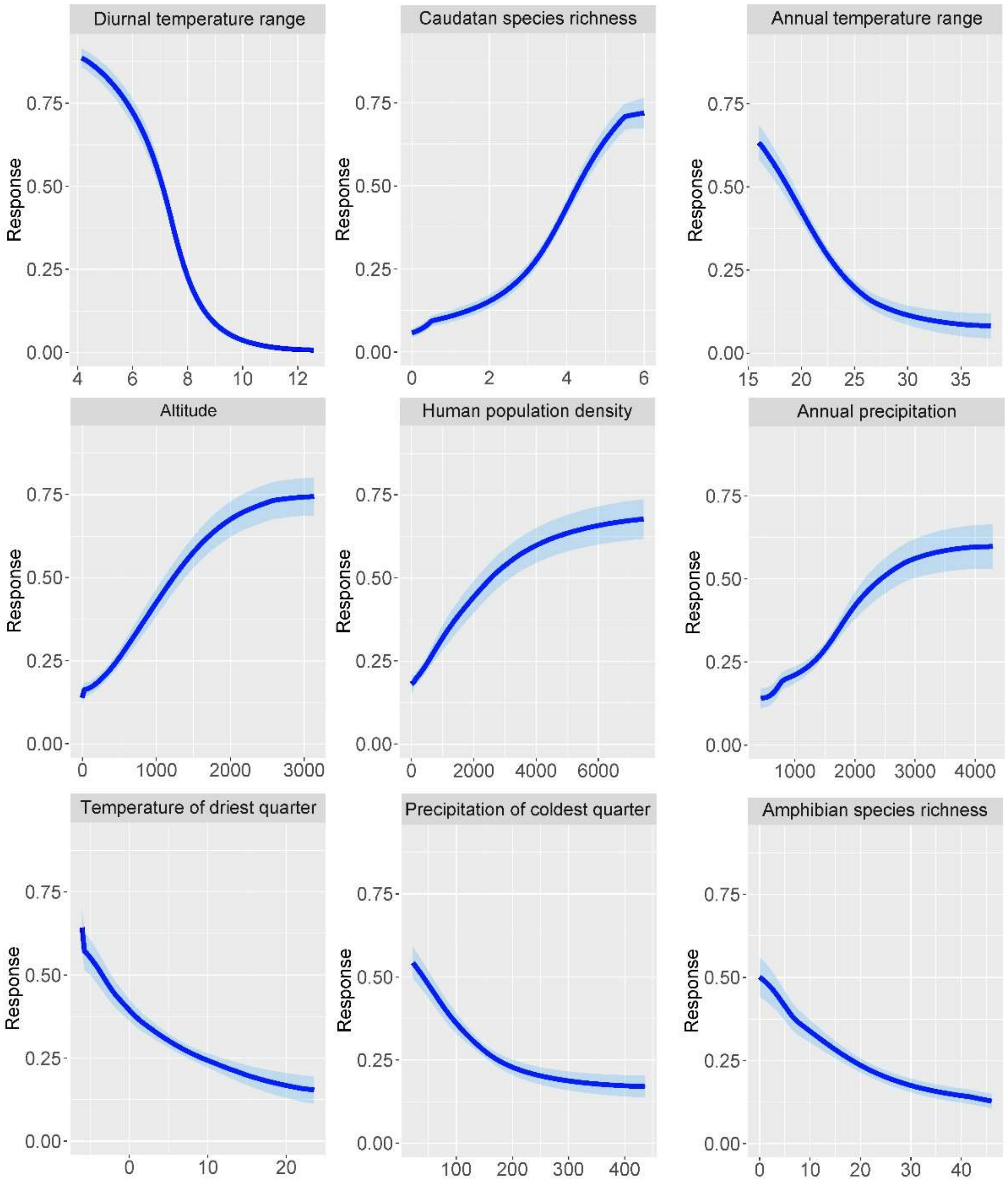

Figure S1. Response of *Bsal* to predictor variables based on the ensemble models. Areas of lower temperature range, more caudate species, high altitude and moderate human population density, were highlghted as suitable habitats for *Bsal*.
